# From trauma to resilience: advancing cultural responsiveness and equity in the Muskowekwan First Nation’s healing journey

**DOI:** 10.3389/fpubh.2024.1419250

**Published:** 2024-08-21

**Authors:** JoLee Sasakamoose, Shauneen Pete, Fred O’Soup, Tiffany Wolfe

**Affiliations:** ^1^Faculty of Education, University of Regina, Regina, SK, Canada; ^2^Interdisciplinary Studies, Royal Roads University, Victoria, BC, Canada; ^3^Muskowekwan First Nation, Lestock, SK, Canada

**Keywords:** historic trauma, cultural responsiveness, Justice, Diversity, Equity, Inclusion (J-DEI), Indigenous healing practices, Indigenous community resilience, mental health and wellness, residential schools in Canada, Indigenous community health

## Abstract

**Introduction:**

The Muskowekwan First Nation (MFN) in Saskatchewan, Canada, epitomizes the enduring strength and cultural preservation of the Saulteaux people. This community faces the lasting effects of colonial trauma, especially the violence, abuse, and adversity experienced by students at the Muskowequan Indian Residential School (MIRS). Decades of abuse by institutional leaders caused generational trauma, contributing to current mental health and well-being challenges. This study highlights the community’s role in sharing experiences and shaping healing processes to develop the MFN Family Healing and Wellness Centre in response to urgent community concerns. It examines the integration of Justice, Diversity, Equity, and Inclusion (J-DEI) principles and cultural responsiveness in fostering community resilience and mental well-being.

**Methods:**

Adopting a community-based participatory research framework, this study employs a mixed-methods approach, including community engagement sessions and surveys. Collaborating closely with the MFN leadership, it draws upon the specialized expertise of Author2 and Author1, leaders in Indigenous health and research. The research uses qualitative and quantitative data collection, emphasizing the importance of community input and leadership in shaping the research process and outcomes.

**Results:**

Findings emphasize the community’s commitment to spiritual and cultural practices as vital healing components. Amidst the heightened awareness of the lingering effects of the MIRS within the MFN community, these insights informed the development of the Centre, ensuring it incorporates the community’s desires for culturally relevant healing practices. The grand opening of Phase I of the Centre in February 2023 emerged as a significant step forward, symbolizing a move towards holistic community health that honors resilience, holistic wellness, and cultural continuity.

**Discussion:**

This case study contributes to the literature on integrated, culturally responsive healthcare models that address the needs of Indigenous peoples and communities. The study provides insights to guide the Centre’s future programs and services, ensuring they are culturally tailored and responsive to the community’s needs. By illustrating the potential for traditional wisdom and contemporary health practices to foster well-being, the case study advocates for holistic approaches to healing in Indigenous settings, offering a replicable framework for similar initiatives globally.

## 1 Introduction

### 1.1 Muskowekwan First Nation: a community contextual analysis

In Saskatchewan, Canada, the Muskowekwan First Nation (MFN) epitomizes the resilience, cultural richness, and spirit of the Saulteaux people. Spanning 16,479 acres near Lestock, Saskatchewan, and recognized as Band 392, this community’s deep-rooted connection to its land was formalized through Treaty 4 in 1874, emphasizing its historical and contemporary significance. As of 2024, MFN comprises 2,173 registered members, with 588 living on the First Nation, reflecting a dynamic balance between historical legacies and modern challenges (1). The historical narrative of the MFN is profoundly marked by the MIRS, which was operational from 1921 to 1997. This institution is a stark reminder of the cultural, linguistic, and identity erosion experienced by many Indigenous communities across Canada, leaving a legacy of intergenerational trauma that continues to reverberate today.

### 1.2 Community initiatives

In response to this legacy, the former Muskowekwan Chief and Council initiated a collaborative effort in 2016 with the Touchwood Agency Tribal Council (TATC) and the Federation of Sovereign Indigenous Nations (FSIN) to establish a Healing and Wellness Centre. Under the research leadership of Dr. Author 1 from Royal Roads University and Dr. Author 2, CIHR Chair in Applied Public Health, Indigenous Wellness at the University of Regina, this research initiative was developed following an invitation from the MFN Chief and Council. The Muskowekwan Family Healing and Wellness Centre, which celebrated its grand opening in February 2023, embodies a forward-looking approach to community health, emphasizing resilience, holistic wellness, and cultural continuity.

### 1.3 Significance of the Healing and Wellness Centre

The envisioned Healing and Wellness Centre is a pillar of hope, providing a sacred space for cultural reconnection, resilience building, and holistic wellness promotion across generations. This center is a tribute to the community’s enduring strength and vitality. It is a model for integrating traditional wisdom with contemporary health practices, empowering individuals and families toward sustained health, spirit, and unity. By presenting the research and development of the Healing and Wellness Centre as a case study, this analysis aims to establish a replicable framework for creating similar centers. This model emphasizes the importance of community-driven engagement, advocating for a system that supports the community’s healing journey while respecting its rich cultural heritage and aspirations.

### 1.4 The enduring impact of historical injustices on Indigenous communities

Historical injustices have perpetuated a cycle of mental health disorders and substance abuse among Indigenous communities in Canada. Research has highlighted the links between historical trauma, adverse childhood experiences (ACEs), and mental health disparities, emphasizing the need for culturally respectful interventions (2–4). Colonization efforts to suppress Indigenous cultures through forced assimilation and family separations have profoundly affected First Nations and Métis peoples’ mental health. The connection between residential school experiences and mental health issues, including distress, suicidal behavior, and intergenerational trauma, is well documented (2, 5).

ACE research underscores the impact of early trauma on lifelong mental and physical health, highlighting the need for early intervention and culturally attuned care (6, 7). Historical traumas correlate with increased psychological distress, depression, anxiety, PTSD, cancer, hypertension, diabetes, and substance abuse (8, 9). The residential school system’s dietary patterns have contributed to higher diabetes rates among Indigenous populations (10). Personal accounts reveal residential school trauma leading to alcoholism, religious disconnection, and relationship difficulties in adulthood (11). Intergenerational trauma amplifies these mental health issues, and the loss of cultural identity and traditional healing practices heightens vulnerability to mental health conditions (4).

### 1.5 The role of the Muskowekwan Family Healing and Wellness Centre

Acknowledging this past and embracing culturally sensitive, trauma-informed approaches are vital for aiding Indigenous communities in their healing and wellness journeys. The Centre is a critical response to these needs, offering a pathway to healing grounded in cultural reconciliation.

These findings highlight the need for targeted, culturally informed interventions at the Centre, addressing mental health disparities rooted in historical trauma. The Centre will provide physical and mental healing while serving as a hub for cultural restoration and community strengthening. By integrating traditional healing practices with contemporary mental health care, the Centre embodies a holistic approach that honors Indigenous cultural heritage. This initiative demonstrates the positive effects of culturally respectful, community-driven health interventions in overcoming historical injustices and promoting holistic wellness in Indigenous communities.

### 1.6 The Muskowekwan Family Healing and Wellness Centre: a paradigm of cultural reconciliation and recovery

Within this context, the Muskowekwan Family Healing and Wellness Centre emerges as an innovative model for Indigenous communities’ mental health and recovery needs. It operationalizes therapeutic landscape concepts and cultural responsiveness, grounding its strategies in the historical and cultural realities of the communities it serves. Informed by the Cultural Responsiveness Framework (CRF) (12, 13) and Indigenous epistemologies, the Centre articulates a holistic healing approach, weaving Indigenous knowledge and practices into its foundational ethos.

### 1.7 Therapeutic landscapes and the path to decolonization

Adopting Gesler’s (14) concept of therapeutic landscapes, the Centre’s design integrates architectural elements inspired by decolonization theories and Indigenous knowledge, including circular layouts and skylights aligned with the four cardinal directions. Such design features helped to associate the land and culture, essential for mitigating colonization’s persistent mental health effects (15, 16). Smith (17) argues that decolonizing healthcare involves the critical task of dismantling colonial structures within treatment approaches to confront the root causes of Indigenous mental health disparities. Marques et al. (18) explore the concept of Therapeutic Cultural Environments (TCE), which meld Indigenous knowledge (Mātauranga Māori) with the natural landscape (19), proposing a culturally attuned framework to promote health and well-being within Māori communities and provide insights into health-and-wellness.

### 1.8 Implementing the cultural responsiveness framework

The cultural responsiveness framework (CRF), co-created with consultation and insights from 74 First Nations communities, guides the Centre’s approach to addressing mental health and substance use challenges. It advocates for revitalizing First Nations health systems and the cultural adaptation of service delivery (13, 20). The Centre’s architectural emphasis on environmental integration and using natural materials exemplifies the CRF’s application, presenting a tangible manifestation of cultural elements in promoting wellness and addressing mental health challenges (12).

### 1.9 Confronting historical trauma through culturally grounded methodologies

Historical trauma and systemic inequities underscore the need for culturally sensitive and equitable health interventions. Community-driven responses that incorporate cultural supports are crucial for reducing morbidity and mortality and addressing social determinants of health (21, 22). The Muskowekwan Centre’s adoption of Justice, Diversity, Equity, and Inclusion (J-DEI) principles represents a significant step toward meeting the health needs of Indigenous peoples. This approach tackles historical injustices, values cultural diversity, ensures equitable access to health resources, and prioritizes Indigenous perspectives in health service planning and implementation.

### 1.10 Land as methodology: reaffirming Indigenous health paradigms

The Centre’s alignment with Indigenous methods, particularly emphasizing the land, respects the MFN’s cultural protocols. By incorporating traditional ceremonies, community narratives, and storytelling, the Centre acknowledges the land’s intrinsic value to Indigenous health, identity, and spirituality (23, 24). The mental health challenges facing First Nations and Métis populations stem from colonization’s traumatic legacies, requiring acknowledgment of historical and systemic injustices. The Muskowekwan Family Healing and Wellness Centre integrates cultural knowledge and ecological mindfulness, offering effective, culturally responsive mental health interventions in Indigenous settings (13, 25).

## 2 Methods

### 2.1 Community-Based Participatory Research approach

Our project used a Community-Based Participatory Research (CBPR) approach, known for effectively addressing public health concerns, advancing social and environmental justice, and empowering individuals to influence their health determinants (26). In collaboration with the Muskowekwan First Nation, we conducted community engagement sessions that were pivotal in creating the Healing and Wellness Centre. Led by Author 1, with support from University of Regina research assistants and Muskowekwan community-based researchers (Authors 3 and 4), our team’s capabilities in community-based research were enhanced. This collaborative effort fostered professional growth and built capacity for community-sensitive engagements (27).

### 2.2 Research questions

The following research questions guided the study:

1. Qualitative Research Questions:

- What are the personal and communal experiences of trauma associated with the Muskowequan Indian Residential School?
- How do community members perceive the long-term impacts of this trauma on their mental health, social relationships, and cultural practices?
- What are the community’s visions and recommendations for healing and wellness?

1. Quantitative Research Questions:

- What are the demographic characteristics of the community members affected by the Muskowequan Indian Residential School?
- How do community members rate their feelings toward family, community, and the natural environment?
- What is the level of engagement and perceived effectiveness of culturally based therapeutic interventions?

### 2.3 Data collection tools

1. Qualitative Data Collection:

- Round Table Discussions: Open-ended questions such as “What happened to you?” and “How do you want to heal?” were used to facilitate in-depth discussions. Research team members and community participants documented responses.
- Narrative Accounts: Participants were encouraged to share detailed personal narratives, which were recorded and transcribed for analysis.

### 2.4 Quantitative data collection

1. Demographic Questionnaire: A structured questionnaire collected demographic data, including age, gender, education level, and relationship to the Muskowequan Indian Residential School (e.g., survivor, descendant).
2. Survey: This survey included questions modified from the Native Wellness Assessment NWA™ (28) and the Awareness of Connectedness Scale (29). It featured Likert-scale questions to assess feelings toward family, community, and the natural environment, as well as the level of engagement in and perceived effectiveness of culturally and spiritually based therapeutic interventions. The Awareness of Connectedness Scale measures the holistic sense of connectedness of the individual with their family, community, and natural environment, an essential element of Indigenous worldviews and a protective factor against substance use and suicide, as well as an aid in recovery from substance use disorders.

### 2.5 Implications for culturally responsive healing framework

The findings from both qualitative and quantitative data are integrated to inform the development of the Family Healing and Wellness Centre. The qualitative insights provided a deep understanding of the community’s experiences and needs, while the quantitative data offered measurable indicators of health and well-being. Together, these data sources illuminate the importance of a culturally responsive healing framework tailored to the specific context of the Muskowekwan First Nation community.

#### 2.5.1 Historical context and research framing

The detrimental impact of the Indian Residential School System on the Muskowekwan First Nation established a vital historical context for our research aims. Guided by the Truth and Reconciliation Commission’s calls to action (2015) and the Tri-Council Policy Statement: Ethical Conduct for Research Involving Humans (TCPS 2), our methodological approach was culturally sensitive, delving into the complex layers of intergenerational trauma and its extensive consequences (30).

#### 2.5.2 Execution of engagement sessions

**Figure 1:**
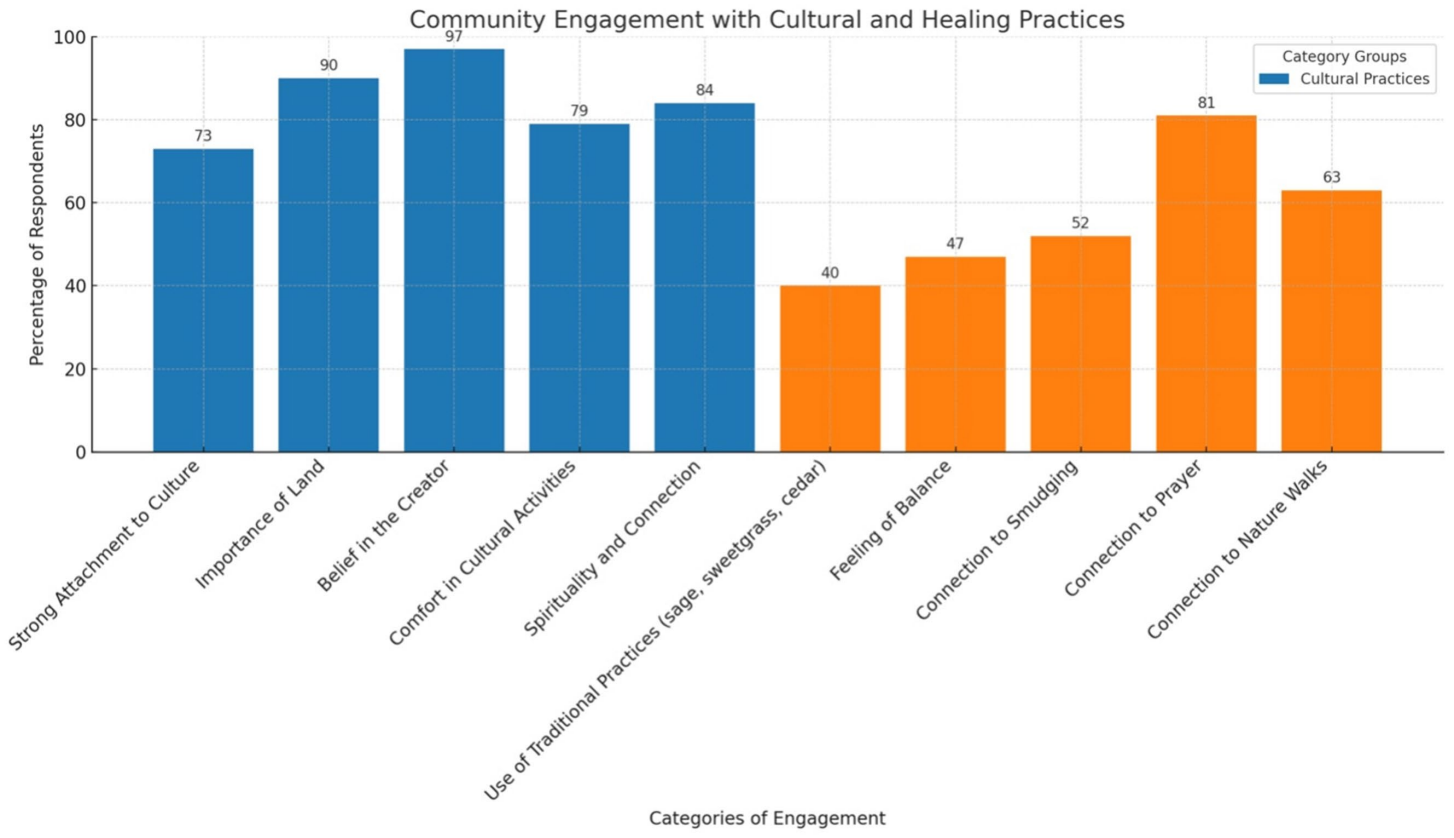
Categories of engagement with cultural healing practices.

Since 18 February 2017, and over 6 years, community engagement sessions have been conducted within the Muskowekwan First Nation. These sessions were developed in collaboration with community leaders and promoted across the region, including First Nations individuals affected by the Muskowequan residential school. The initial session set the groundwork for future sessions and gathered foundational data. Traditional prayers initiated the session, fostering an atmosphere of respect and openness. Approximately 50 participants, including elders, knowledge keepers, youth, residential school survivors and their descendants, and other community members, provided comprehensive perspectives on the community’s experiences and needs (Figure 1).

#### 2.5.3 Study design overview

The session was structured into two main parts:

1. Exploring the trauma:

- Participants were asked, “What happened to you?” allowing them to share personal experiences and narratives about the trauma endured, mainly focusing on the violence and abuse at the Muskowequan Indian Residential School. This discussion took the entire morning, ensuring ample time for sharing in a supportive environment. Educational psychology students on the research team provided emotional support.

1. Designing the healing process:

- Following lunch, participants completed a questionnaire on demographics and feelings toward family, community, and cultural interventions. The discussion then resumed with, “How do you want to heal from it?” gathering insights and suggestions on their visions for healing and how the Family Healing and Wellness Centre could support this process.

#### 2.5.4 Detailed process and discussion starters

Counselor Cindy Desjarlais welcomed participants, providing background information about the collaboration between FSIN, TATC, Muskowekwan, and the research team. Drs. Pete and Sasakamoose introduced themselves and the research assistants, explaining the session’s purpose and format. During round table discussions, research team members, including educational psychology students, sat with participants to offer support, recognizing the challenges of discussing personal and community traumas. Responses were documented and reviewed with the community for accuracy and additional input.

### 2.6 Data collection and analytical strategy

Our approach to engagement facilitated thorough and iterative discussions with community members. Feedback from sessions was analyzed and integrated into conference-style posters, fostering continuous community involvement. Descriptive statistical analysis examined the quantitative data, while thematic analysis was used for qualitative data. This dual approach provided an in-depth understanding of the community’s priorities and requirements, influencing the Healing and Wellness Centre’s design and construction.

### 2.7 Ethical considerations and participant engagement

Given the project’s sensitive nature, ethical rigor was paramount, emphasizing respect for Indigenous knowledge, cultural protocols, and historical injustices (31). The study received ethical approval from the appropriate review board, and all participants provided informed consent. An iterative feedback mechanism stressed the CBPR tenets of reciprocity, respect, and genuine community partnership (32). Our approach honored the Ownership, Control, Access, and Possession (OCAP)™ principles, affirming the community’s sovereignty over their data.

### 2.8 Study design and inclusivity

The study focused on understanding the trauma experienced by those who attended the MIRS, recognizing it as a critical source of historical and current community trauma. MFN community leadership included diverse members affected by the school’s presence to capture the broader intergenerational impacts. Central to our methodology was integrating traditional knowledge and practices, aligning our research with community values, and cementing the efficacy of the CBPR approach. Over 6 years, this project engaged the First Nation community in sessions pivotal to developing the Centre. Led by the principal investigator, with support from university research assistants and community-based researchers, these sessions enhanced the team’s community-based research capabilities and facilitated professional growth.

## 3 Data analysis

We employed a multifaceted qualitative method grounded in thematic analysis, enriched by grounded theory principles, allowing themes to emerge naturally from the data (33). This was augmented by content analysis to explore textual data for recurring patterns, meanings, and relationships (34). Hierarchical coding techniques structured our analysis, organizing data into themes and sub-themes reflecting the community’s experiences and insights. A mind map diagram visually represented our findings, enhancing accessibility and interpretability (35).

### 3.1 Identification of themes

**Figure 2:**
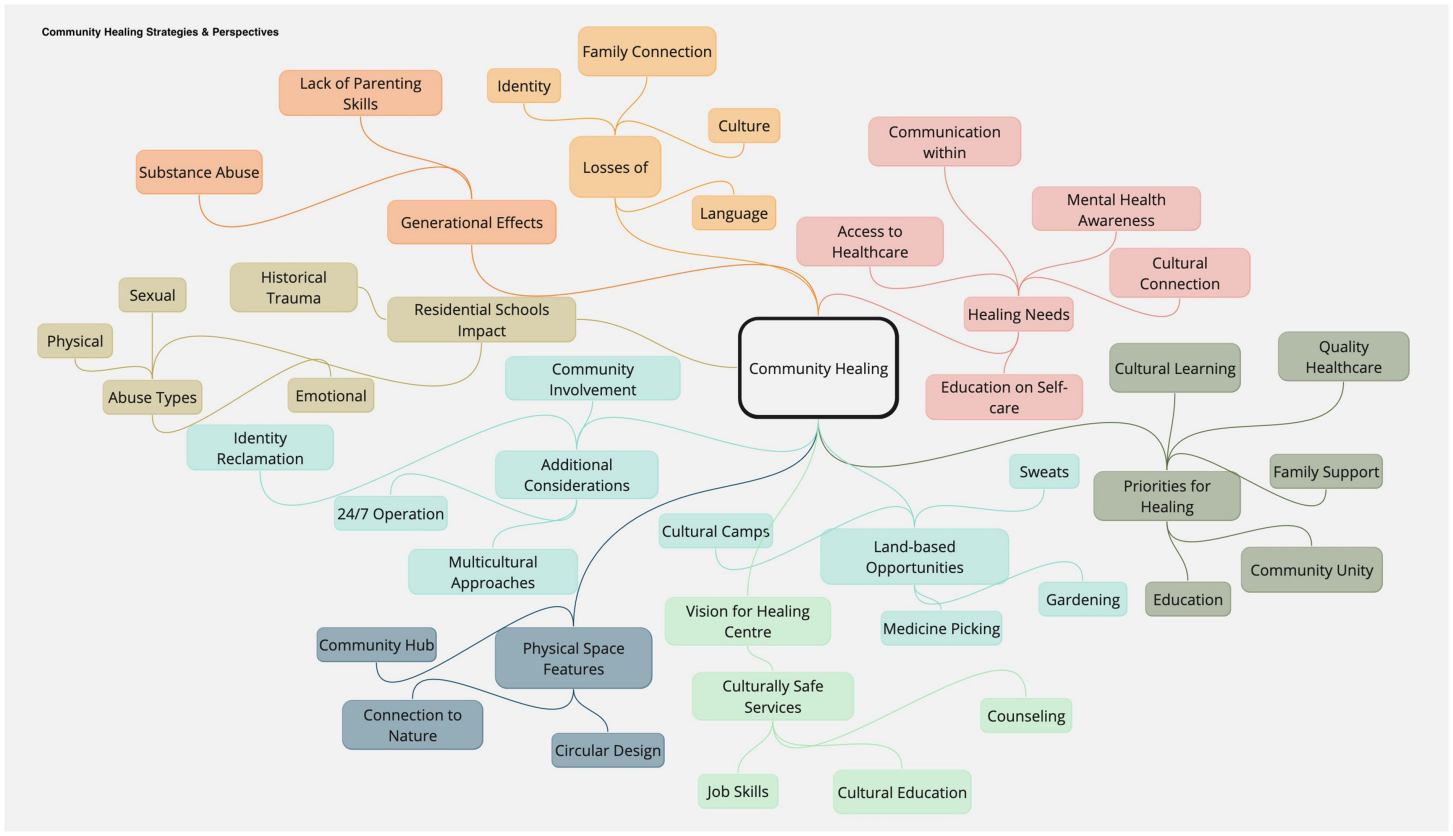
Community healing strategies and perspectives.

A thorough review of the qualitative data highlighted themes like the impact of historical trauma, community healing needs, and visions for the healing center. This iterative process aligned with grounded theory’s emphasis on data-driven category development (36). Detailed examination of participant responses distilled sub-themes, providing a granular view of the community’s perspectives, such as “Residential School Effects” and “Intergenerational Consequences,” each supported by direct participant quotes. Specific points highlighted by community members were outlined within each sub-theme, capturing the depth of the community’s experiences and the multifaceted nature of historical trauma (Figure 2).

### 3.2 Structural clarity in mind map

Themes, sub-themes, and specific points were organized in a hierarchical mind map, visually representing complex data illustrating relationships between broad themes and particular insights.

### 3.3 Incorporating emotional and ethical reflections

The emotionally charged data required a sensitive approach, incorporating “two-eyed seeing” or Etuaptmumk, which uses both Indigenous and Western perspectives to emphasize empathy and ethical responsibility (37). Reflecting on the emotional impact of historical trauma narratives, our methodologies respected the community’s voice and adhered to ethical standards in Indigenous research (17). Through this reflective process, we documented the Muskowekwan First Nation’s journey toward healing and contributed to the discourse on culturally responsive methodologies in Indigenous health research. Our approach integrated traditional knowledge with qualitative research practices, representing the community’s experiences, challenges, and aspirations for healing and wellness.

### 3.4 Applying the cultural responsiveness framework

The CRF within the Muskowekwan First Nation and the broader Indigenous community in Saskatchewan demonstrated impacts on healing and restoration, surpassing initial expectations. Our research extended beyond traditional academic pursuits to embrace healing the land and its people, marked by the legacies of ancestors and children. Genuine engagement with the community’s stories of sorrow and resilience marks a crucial phase in their trauma recovery and healing journey. This reflective journey reinforces the critical need for research that adheres to ethical standards and resonates with Indigenous communities’ emotional and cultural realities. It highlights the transformative potential of embracing culturally grounded methods and the essential role of empathy in navigating the complexities of Indigenous health research.

## 4 Findings

This case study reveals the Muskowekwan First Nation’s significant progress towards healing and well-being, anchored in its rich cultural practices and traditional healing methods. Employing a mixed-methods approach, this case study provides insights into these practices’ critical role in the community’s collective path to healing.

### 4.1 Quantitative insights

Data indicates a high level of engagement within the community with spiritual and cultural beliefs. Approximately 97% of participants acknowledged the importance of belief in the Creator, and 90% emphasized the connection to the land for their well-being. Participation in cultural activities (79%) and spiritual practices (84%) demonstrates the community’s dedication to maintaining spiritual health and fostering a sense of belonging.

#### 4.1.1 Diversity and nuances in traditional healing practices

The community’s engagement with traditional healing practices showcases a spectrum of activities. While prayer (81%) and nature walks (63%) emerge as common practices, using sage, sweetgrass, and cedar is less prevalent, with 40% of respondents incorporating these elements into their healing routines. This variation suggests a rich tapestry of healing practices within the community, each carrying its significance and pointing to areas where community knowledge and access to traditional materials could be enhanced.

#### 4.1.2 Implications for community healing

The quantitative outcomes reveal the pivotal role of cultural and spiritual engagement in the community’s healing journey. By engaging in various spiritual and cultural practices, community members foster a sense of identity, belonging, and well-being, highlighting the importance of these elements. The diversity in traditional healing practices reveals the community’s rich cultural heritage. It suggests opportunities for further enriching this aspect of communal life through educational initiatives and increased support for accessing and preserving traditional healing materials and knowledge.

### 4.2 Qualitative insights

Discussions with the MFN about the healing and wellness center revealed key themes:

#### 4.2.1 Historical trauma and its legacy

Participants’ narratives illustrated the lasting scars from the residential school system, highlighting the urgent need for healing initiatives that address psychological impacts and foster reconnection with lost cultural identities and practices.

#### 4.2.2 Priority for holistic healing approaches

There is consensus on the need for holistic healing that addresses mental, physical, emotional, and spiritual health. This aligns with Indigenous views on health and wellness, reflecting a desire to integrate traditional healing practices with contemporary health interventions.

#### 4.2.3 Cultural safety and land-based healing

Participants emphasized the importance of land and cultural practices in healing, expressing a desire for spaces that respect traditional values and aim to reclaim and revitalize land-based healing practices. Integrating these insights with quantitative data provides a comprehensive view of the Muskowekwan First Nation’s healing journey, underscoring a community drawing strength from its cultural roots while navigating historical traumas. The collective aspiration for a holistic, culturally grounded approach to wellness emphasizes the need for policies that honor Indigenous healing practices. The Family Healing and Wellness Centre exemplifies the community’s commitment to intertwining healing with cultural traditions and collective history, offering valuable insights for other communities seeking healing and cultural reclamation.

### 4.3 Fulfilling the vision: Muskowekwan Family Healing and Wellness Centre takes shape

The MFN is constructing a $2 million Family Healing and Wellness Centre, funded by stakeholders including Indigenous Services Canada, to address the historical effects of residential schools, the Sixties Scoop, and other traumatic events.

#### 4.3.1 The urgent need for healing

Former Chief Reginald Bellerose emphasizes the urgency of this project in addressing historical traumas. The Centre aims to foster an environment where families in crisis can come together and embark on a collective healing journey.

#### 4.3.2 Community-driven approach

The Centre is conceived and propelled “by the community for the community,” aiming to create a welcoming, homelike environment where families can find the support they need to heal collectively.

#### 4.3.3 A model informed by cultural responsiveness

Rooted in the Cultural Responsiveness Framework, the Centre addresses the systemic and long-term effects of historical trauma from the residential schooling system and the Sixties Scoop in Canada.

#### 4.3.4 Holistic healing for families

The Centre focuses on healing families rather than individuals, integrating various health, mental health, and wellness services. It emphasizes cultural and traditional support, mainly land-based programming.

#### 4.3.5 Extended in-residence healing

The Centre will house families for extended periods, prioritizing patient- and family-centered outcomes with trauma-informed care, wrap-around support, and multidisciplinary practitioners.

#### 4.3.6 Guiding principles and cultural integration

Guided by community engagement sessions, the design integrates cultural nuances, with the circle symbolizing tradition and cultural significance. The center uses natural materials and aims for a “feel like home” ambiance.

#### 4.3.7 Visualizing healing spaces

Conceptual renderings show family dwelling units facing a central circle, supporting outdoor gatherings and cultural events. An intimate gathering circle facilitates family healing ceremonies, discussions, and cultural practices.

The Muskowekwan Family Healing and Wellness Centre is more than a construction project; it is a testament to resilience, cultural revival, and a community’s commitment to healing, shaping a future that honors its past.

## 5 The painful discovery of unmarked graves: a solemn tribute and ongoing investigation

Global media have covered stories about First Nations and the legacy of residential schools. Honoring the community’s oral history was crucial for building a new Healing and Wellness Centre on MFN land, revealing unmarked graves of children and adults from the residential school. Collaborative efforts between the MFN, University of Saskatchewan, and University of Alberta using ground-penetrating radar in 2018 and 2019 identified at least 35 graves (38). Earlier water line construction in the 1990s suggested more graves might await discovery. Elders believe unexplored areas remain, a task delayed due to funding challenges and the COVID-19 pandemic. The Federation of Sovereign Indigenous Nations (FSIN) and the Saskatchewan government are advocating for federal support to fund further radar searches at residential schools. FSIN Chief Bobby Cameron praised Saskatchewan’s proactive stance in organizing these searches, stating, “The Muskowekwan community continues its path to healing and building upon resiliencies from enduring Canadian residential school trauma.”

In acknowledgment of the residential schools’ dark legacy, the MFN laid out 35 pairs of children’s moccasins and shoes at the former residential school site, each pair symbolizing an unmarked grave discovered during investigations in 2018 and 2019. The ceremony also honored the 215 children found at Kamloops residential school and the 751 at Cowesses First Nation’s Marieval Residential School.

During a somber gathering, Brian Wolfe [pseudonym], who spent 9 years at the Muskowekwan Indian Residential School, shared his experience. Taken from his family at seven, he recalls the experience of being separated from his family. Despite living mere meters away, he was forbidden to rejoin his family. Recounting instances of enjoyment, such as playing sports and hardship, enduring physical abuse for trying to learn his language, Wolfe reflects on the lost opportunity to inherit his father’s Cree language.

I missed out on learning my father’s Cree language. It’s like a piece of my identity is missing, Wolfe shared, grieving the cultural connections lost to him.

Jimmy Sayer attended the MFN residential school from 1983 to 1984, and he articulates his experience.

I’ve spent half my life incarcerated, and I blame the residential school for that. But I also know I must give up my hate because I’m responsible for myself. I have three adult daughters, and I was in jail for the duration of their childhoods. I have a two-year-old son now, and I need to be there for him; I have to be different.

The discoveries of unmarked graves at the site of the former residential school on MFN territory starkly remind us of the painful legacies that persist within Indigenous communities. This solemn tribute and ongoing investigation unearth truths that have long been silenced, igniting a collective resolve to honor those lost and support the healing journeys of survivors. The collaborative efforts to map out and reclaim these sacred spaces are acts of resilience and restoration, reaffirming the community’s commitment to remembering and learning from the past. As we look to the future, we must continue to support these endeavors, ensuring that the voices of the community guide us and that we never forget the profound impacts of these historical injustices. Through remembrance, acknowledgment, and informed action, we contribute to the healing and strengthening of the community, paving the way for a future where such injustices are no longer repeated.

### 5.1 Moving towards reconciliation: a symbolic designation and commitment to truth

Acknowledging the residential school system as a shameful and racist colonial policy, as stated in the government’s news release, marked a critical step towards reconciliation. Designating the former school as a national historic site aligned with Canada’s commitment to the Truth and Reconciliation Commission’s calls to action, particularly addressing call 79. This call urges implementing a national heritage plan and strategy to commemorate residential school sites and their histories.

Former Chief Reginald Bellerose of the MFN emphasized the significance of this designation, expressing hope for a new era of reconciliation and learning. The national historic site stands as a solemn reminder of the enduring negative impacts of residential schools on Indigenous communities, cultures, and ways of life. The MFN can now share its truth and rewrite a narrative long overlooked in the broader Canadian historical context.

## 6 Discussion: implications, lessons learned, and embracing land-based healing across spiritual practices

This investigation into the Muskowekwan Family Healing and Wellness Centre demonstrates the Muskowekwan First Nation’s commitment to healing and wellness, rooted in cultural and spiritual practices. Through integrating quantitative and qualitative research methodologies, the study has illuminated the significant role of cultural and spiritual engagement in the community’s healing journey. It has highlighted how the connection to land and the embrace of land-based healing practices provide a unifying foundation across the diversity of spiritual beliefs, including traditional Indigenous spirituality and Christianity.

### 6.1 Cultural and spiritual engagement

The data showcases a diverse community where spiritual practices and beliefs reflect a rich tapestry of traditional Indigenous spirituality alongside Christianity. Despite this diversity, land-based healing is a common thread that unites these varying practices. The Muskowekwan Family Healing and Wellness Centre, guided by the Cultural Responsiveness Framework (CRF), navigates this diversity by focusing on land as a central element in healing, respecting the deep-rooted connection that traditional and Christian community members feel towards the land.

### 6.2 Embracing land-based healing

The emphasis on land-based healing practices presents a unique opportunity to bridge traditional Indigenous and Christian spiritual approaches. This focus acknowledges the land as a source of healing, wisdom, and identity for the Muskowekwan community, transcending individual spiritual beliefs. By grounding healing interventions in the land, the center fosters an inclusive environment where all community members, regardless of their spiritual orientation, can find common ground and support in their healing journeys.

### 6.3 Challenges and opportunities

Integrating diverse spiritual practices focusing on land-based healing brings challenges and opens pathways for meaningful community engagement and healing. Key lessons from the MFN Family Healing and Wellness Centre include:

- Unified Healing Approach: Successful healing practices recognize the unifying power of the land. Emphasizing land-based interventions can transcend the diversity of spiritual beliefs within Indigenous communities, offering a shared space for healing and connection.
- Inclusive Community Engagement: Involving the community in developing and initiatives ensures diverse spiritual practices are respected and integrated, with land-based healing serving as a common platform for engagement.
- Educational and Dialogical Initiatives: Educational programs play a valuable role in highlighting the significance of land in healing practices. Creating spaces for dialog about the intersections of land, spirituality, and healing can enhance understanding and respect among community members who follow different spiritual paths.

### 6.4 Toward a culturally responsive healing framework

This study underscores the importance of culturally congruent health interventions that honor the land’s role in Indigenous healing. The Muskowekwan First Nation’s experience illustrates how healthcare models can embrace a holistic approach, using land-based healing to bridge diverse spiritual beliefs. The Muskowekwan Family Healing and Wellness Centre is a model for creating inclusive healing spaces that accommodate Indigenous spiritual and cultural diversity, promoting unity, resilience, and wellness.

### 6.5 Future directions

Future research could explore the long-term impacts of culturally grounded health programs, providing a replicable model for other Indigenous communities. Key areas of focus include:

- Expanding participatory research to include a broader spectrum of Indigenous communities, enriching the analysis of healing practices.
- Investigating the implementation and efficacy of policy and practice changes, particularly those emphasizing J-DEI principles.

These insights informed the development of the Family Healing and Wellness Centre, ensuring that services align with the community’s needs and preferences for culturally integrated healing practices.

### 6.6 Policy and practice implications

This study highlights the need for policies prioritizing Indigenous healing traditions within broader health initiatives. Recommendations include:

- Formal recognition and integration of traditional healing practices within public health frameworks.
- Implementing cultural safety training for healthcare providers to enhance respectful and inclusive services.
- Allocating resources to support language and cultural revitalization programs as integral to mental health and wellness initiatives.
- Developing land-based healing programs that align with Indigenous perspectives on health and well-being.

Incorporating these recommendations can create a healthcare landscape that addresses the unique needs of Indigenous communities while celebrating and preserving their rich cultural heritage. This study contributes to the discourse on Indigenous health, highlighting the transformative potential of culturally responsive healing initiatives in fostering resilience, well-being, and vibrant community life for the Muskowekwan First Nation and beyond.

## Data availability statement

The datasets presented in this article are not readily available because in alignment with the OCAP® principles that govern the rights of Indigenous communities to own, control, access, and possess information about themselves, the datasets generated and analyzed during this study are not publicly available. The Muskowekwan First Nation, as the custodian of the data, has determined that the sensitive nature of the mental health information and the small community size necessitate stringent confidentiality measures to protect individuals’ anonymity and privacy. Given the intimate connection between the data and the community’s well-being, coupled with the ethical considerations surrounding mental health research, all data remains under the direct stewardship of the Muskowekwan First Nation. Access to the data is restricted to authorized personnel approved by the community, following agreed-upon protocols that respect the community’s sovereignty and the individual participants’ confidentiality. For researchers interested in understanding more about the methodologies employed or the insights garnered from this study in a manner that respects these constraints, we encourage direct communication with our corresponding author. We are committed to sharing knowledge and best practices derived from our work with the Muskowekwan First Nation in ways that honor and uphold the principles of OCAP® and the community’s requirements. Further information on the ethical guidelines and community agreements that underpin this study’s data management practices can be found in the “Availability of Data” section of our Materials and Data Policies, as outlined in the Author Guidelines. These policies have been developed to ensure that our research practices comply and actively support the rights and responsibilities articulated through OCAP®. Requests to access the datasets should be directed to jolee.sasakamoose@uregina.ca.

## Ethics statement

The studies involving humans were approved by University of Regina Ethics Board. The studies were conducted in accordance with the local legislation and institutional requirements. Written informed consent for participation in this study was provided by the participants.

## Author contributions

JS: Conceptualization, Data curation, Formal analysis, Funding acquisition, Investigation, Methodology, Project administration, Resources, Software, Supervision, Validation, Visualization, Writing – original draft, Writing – review & editing. SP: Conceptualization, Data curation, Formal analysis, Investigation, Methodology, Project administration, Resources, Supervision, Validation, Writing – review & editing, Writing – original draft. FO'S: Data curation, Formal analysis, Investigation, Validation, Writing – review & editing, Writing – original draft. TW: Data curation, Formal analysis, Investigation, Validation, Writing – review & editing, Writing – original draft.
